# HIV-1, Methamphetamine and Astrocyte Glutamate Regulation: Combined Excitotoxic Implications for Neuro-AIDS

**DOI:** 10.2174/157016212802138832

**Published:** 2012-07

**Authors:** Irma E Cisneros, Anuja Ghorpade

**Affiliations:** University of North Texas Health Science Center, Fort Worth, TX, USA

**Keywords:** Astrocytes, EAAT-2, excitotoxicity, GFAP, glutamate, human immunodeficiency virus-1, methamphetamine.

## Abstract

Glutamate, the most abundant excitatory transmitter in the brain can lead to neurotoxicity when not properly regulated. Excitotoxicity is a direct result of abnormal regulation of glutamate concentrations in the synapse, and is a common neurotoxic mediator associated with neurodegenerative disorders. It is well accepted that methamphetamine (METH), a potent central nervous stimulant with high abuse potential, and human immunodeficiency virus (HIV)-1 are implicated in the progression of neurocognitive malfunction. Both have been shown to induce common neurodegenerative effects such as astrogliosis, compromised blood brain barrier integrity, and excitotoxicity in the brain. Reduced glutamate uptake from neuronal synapses likely leads to the accumulation of glutamate in the extracellular spaces. Astrocytes express the glutamate transporters responsible for majority of the glutamate uptake from the synapse, as well as for vesicular glutamate release. However, the cellular and molecular mechanisms of astrocyte-mediated excitotoxicity in the context of METH and HIV-1 are undefined. Topics reviewed include dysregulation of the glutamate transporters, specifically excitatory amino acid transporter-2, metabotropic glutamate receptor(s) expression and the release of glutamate by vesicular exocytosis. We also discuss glutamate concentration dysregulation through astrocytic expression of enzymes for glutamate synthesis and metabolism. Lastly, we discuss recent evidence of various astrocyte and neuron crosstalk mechanisms implicated in glutamate regulation. Astrocytes play an essential role in the neuropathologies associated with METH/HIV-1-induced excitotoxicity. We hope to shed light on common cellular and molecular pathways astrocytes share in glutamate regulation during drug abuse and HIV-1 infection.

## 1 INTRODUCTION

Methamphetamine (METH) is a psychostimulant that results in strong, long-lasting, euphoric effects. METH heightens the libido and impairs judgment, which can lead to risky sexual behavior, increasing an individual’s risk for acquiring human immunodeficiency virus (HIV-1). HIV-1 is a virulent and infective virus that causes progressive immune system failure, replicating rapidly in the periphery during acute infection. There was an estimated 34 million patients living with HIV-1 at the end of 2010. Accurate statistics documenting the numbers of HIV-1 infected individuals who are METH abusers are not available; however, the prevalence of HIV-1 infections with METH abuse is high. Thus, mechanisms resulting in combined clinical outcomes of the two are of significant importance. Neurotoxicity is a critical consequence of both METH abuse and HIV-1 infection. HIV-1 results in cognitive deficits collectively referred to as HIV-associated neurocognitive disorders (HAND), HIV-associated dementia (HAD) as the most severe form. Chronic METH abuse is associated with neuropsychiatric complications including deficits in attention, memory, and executive functions and leads to the degeneration of dopaminergic neurons responsible for deteriorating behavioral processes such as mood, addiction and reward, and exacerbates HAD [1]. Combined effects of METH and HIV-1 include excitotoxicity, oxidative stress, glial cell activation, inflammation and hyperthermia; together leading to neurotoxicity [2,3]. Studies indicate that METH dependence has an additive effect on neuropsychological deficits associated with HIV-1 infection [4]; however, direct mechanisms involved in METH/HIV-1-induced combined neurotoxicity remain incompletely defined. METH/HIV-1-induced glial cell activation is of specific importance due to the abundance of astrocytes in the central nervous system (CNS) and their contribution to excitotoxicity, a common mechanism in neurodegenerative and neurotoxic disorders including METH/HIV-1 combined injury. Extracellular levels of glutamate in the CNS microenvironment are tightly regulated *via *glutamate transporters expressed predominantly on astrocytes and excitotoxicity is a direct result of brain glutamate dysregulation [5]. During homeostasis, rapid removal of glutamate in the synapse *via *these transporters ensures optimal glutamate balance, while in brain injury or disease, glutamate transporter expression and function may be impaired. Moreover, in such conditions, transporters can also work in reverse, boosting extracellular glutamate accumulation in the synapse [6,7]. In addition to regulating glutamate levels, expression of neuronal glutamate receptors and astrocyte metabotropic glutamate receptors (mGluRs) is also influenced by glutamate transporter function. The balancing of glutamate synthesis and degradation, for which astrocytes possess both necessary enzymes, regulates the duality between glutamate levels and receptors [8-10]. Other neurotoxic mechanisms induced by METH and HIV-1; such as astrogliosis, blood brain barrier (BBB) impairment, excitotoxicity, and inflammation, are likely implicated in intracellular signaling events by which astrocytes modulate glutamate uptake, synthesis and metabolism.

## 2 ROLE OF ASTROCYTES IN THE CENTRAL NERVOUS SYSTEM

Astrocytes support endothelial cells and are juxtaposed across them at the BBB. The ability of neurotoxic mediators to cross the BBB activates astrocytes directly, modulating their inflammatory profiles both at the BBB, as well as in brain parenchyma. Proinflammatory cytokines tumor necrosis factor (TNF)-α, interleukin (IL)‐1β, and IL‐6 disrupt endothelial cell function *via *astrocyte activation [11] allowing the passage of otherwise restricted substances into the brain. Chaitanya *et al.* treated endothelial cell monolayers and endothelial cell/astrocyte cocultures with proinflammatory cytokines, TNF-α, IL-1β, and interferon (IFN)-γ [11]. The cytokines did not reduce BBB integrity directly through interaction with endothelial cells; however, in the coculture model the presence of astrocytes significantly reduced barrier integrity. These data suggest that the gliovascular unit comprised of astrocytes and associated microvasculature, senses changes in local metabolism and physiological unbalance during pathological and inflammatory conditions. The primary response to inflammatory mediators occurs through astrocytes, indicating that their activation leads to the aggravation and dysfunction of the gliovascular unit, subsequent impairment of the BBB and ultimately neuronal dysfunction. Proinflammatory cytokines increase BBB permeability by regulating microfilament reorganization, nuclear factor (NF)-κB activation and differential expression of tight junction (TJ) proteins [12,13]. Proinflammatory cytokine-mediated release of matrix metalloproteinases (MMP)-9 and -13 further alters BBB permeability through remodeling and degradation of extracellular matrix proteins (ECM) [14,15]. Furthermore, astrocytes are the primary source of tissue inhibitors of MMPs (TIMPs) in the brain [16,17]. Dysregulation of the MMP:TIMP-1 balance alters ECM remodeling and degradation, and may eventually inhibit neuronal regeneration [16,18,19]. Chronic astrocyte activation escalates the disruption of endothelial cells and eventually results in endothelial cell apoptosis through the dysregulation of lipoxygenase and cyclooxygenase (COX), large/big calcium (Ca^+2^)-activated potassium (K^+^) channels, and ATP receptor activation within astrocytes [20].

Astrocyte activation upregulates glial fibrillary acidic protein (GFAP) as exhibited by increased cellular migration, extension of astrocyte processes and excessive proliferation. GFAP, an intermediate filament protein, is involved in cell communication, mitosis, cell migration, and cytoskeletal changes. GFAP upregulation is a hallmark feature observed in neurodegenerative disorders such as Alzheimer’s disease, Parkinson’s disease, and HAD [21]. Lu *et al.* assessed reactive astrocytic intermediate filament expression in respect to the rigidity of glial scars impairing neuronal regeneration in mouse retina. The upregulation and stiffness of GFAP and vimentin was prominent within the endfeet and inner stem processes of reactive astrocytes and significantly contributed to inhibition of neuronal regeneration. In other studies, suppression of intermediate filament upregulation, by GFAP small interfering RNA (siRNA), improved synaptic and neuronal regeneration [22]. The mechanism resulting in GFAP upregulation is unclear, but promoter studies of the 5’ flanking region of the human and mouse GFAP genes (gfa2) demonstrated transcriptional activity increased 75-fold upon the insertion of three additional GFAP enhancer regions [23]. The transcription factor family nuclear factor of activated T-cells (NFAT) is implicated in the activation of astrocytes responding to inflammatory mediators IL-1β, TNF-α, ATP and glutamate [24-26] leading to increased expression of COX-2 and TNF-α affecting neuronal cells in close juxtapositions [27]. NFAT activation requires translocation from the cytoplasm to the nucleus and is triggered by an intracellular Ca^+2^/calmodulin-dependent phosphates, which links Ca^2+^ signaling and NFAT-dependent gene transcription in astrocytes [28].

Ca^+2^ is a vital second messenger in astrocytes and is involved in astrocyte responses and communication. Brain network of astrocytes is linked *via *gap junctions that transmit Ca^+2^ waves [29]. Studies of gap junction protein, connexin (Cx) 43, localized intensively along astrocytic end processes demonstrate ATP-induced mobilization of cytosolic Ca^+2^ to end feet with an electrical stimulated release of Ca^+2^ [30]. Gap junctions are intercellular junctions formed in adjacent cells linking the cytoplasm of the two cells directly, which facilitates direct transfer or ions. In addition to ions, gap junctions also transfer small molecules such as chemokines [20]. Increases in intracellular Ca^+2^ concentration [Ca^+2^]_i_ and inositol phosphate (IP_3_) are also associated with diffusion through gap junctions from adjacent cells [31] as well as astrocyte metabotropic and ionotropic glutamate receptor (iGluR) activation propagating intracellular Ca^2+^ waves [32]. James *et al. *compared intracellular Ca^+2^ propagation upon activation of mGluRs and iGluRs by glutamate, histamine, and ATP stimulation in astrocytes [32]. These agonists represent physiological stimuli; an excitatory transmitter, an inhibitory transmitter and an inflammatory messenger, respectively, which result in Ca^+2^ signaling. While increases in [Ca^+2^]_i_ levels were not significantly different upon activation of mGluRs and iGluRs by ATP, glutamate and histamine, the Ca^+2^ responses; such as single spikes, bursts of spikes, repetitive oscillations or sustained increases in Ca^+2^, were different but not robust. Neurotransmitters and other excitatory molecules, glutamate, D-serine and ATP, are Ca^2+^-regulated as well, further implicating a critical role of Ca^+2^-regulation in astrocyte function and synaptic transmission in the CNS [33-35]. The role of astrocytes in synaptic plasticity, release of gliotransmitters, chemokines (CCL2, IL-1β), neuronal growth factors, protection against oxidative stress along with Ca^+2^-regulated events, implicates a critical contribution of astrocytes to neuroprotection in the CNS microenvironment.

## 3 ROLE OF GLUTAMATE IN THE CENTRAL NERVOUS SYSTEM

Glutamate is an essential excitatory neurotransmitter vital for cognition, learning and memory and is critical for long-term potentiation (LTP) and synaptic plasticity *via *ligation of glutamate receptors leading to intracellular responses [5]. In the brain, approximately 40% of all neuronal synapses are glutamatergic, thus regulation of glutamate concentrations is crucial for optimal glutamatergic neurotransmission [36]. Rapid uptake of glutamate *via *astrocyte glutamate transporters is responsible for up to 90% of extracellular brain glutamate uptake, and thus promotes termination of excitatory signals [37]. Astrocytes possess enzymes responsible for glutamate-glutamine conversion. Further, the pool of glutamine is transported to adjacent neuronal cells serving as a source of glutamate for neurotransmission [37]. Approximately 99.99% of glutamate is stored intracellularly, where it remains inactive preventing deleterious neurotoxic effects [38].

Accumulation of extracellular glutamate and subsequent overstimulation of glutamatergic receptors disrupts neuronal [Ca^+2^]_i_ homeostasis, which induces increased reactive oxygen/nitrogen species (ROS/NOS) levels [39]. Dysfunctional glutamate transporters and resultant increased glutamate levels are implicated in epilepsy, stroke and other neurodegenerative diseases, as well as, in neuroinflammation and HAD [40].

### 3.1 Transporters and Receptors

Two distinct classes of glutamate transporters regulate glutamate transport across cell membranes: excitatory amino acid transporters (EAAT) and vesicular glutamate transporters (VGLUT). EAATs scavenge glutamate from synaptic space and convert it to glutamine as the part of the glutamate/glutamine shuttle. EAATs are dependent on the electrochemical gradients of sodium (Na^+^) and K^+^ ions [38]. VGLUTs, in contrast, pack glutamate into vesicles to be released back into the synapse as neurotransmitters independent of the electrochemical gradient [41]. Both in neurons and astrocytes, glutamate receptors are responsible for the glutamate-mediated excitation of neural cells and are essential for neural communication and LTP. To date two types of glutamate receptors have been identified in astrocytes, each with high affinity for glutamate [5]. iGluRs form an ion channel pore, which is activated upon glutamate binding. mGluRs activate cationic ion channels through G-protein coupled receptors (GPCRs) and intracellular signaling. Fig. (**1**) illustrates the glutamate transporters, receptors, and enzymes as localized on astrocytes. How each is implicated in glutamate regulation is discussed below.

#### 3.1.1 Excitatory Amino Acid Transporters

To date, five cloned subtypes of EAATs are described that regulate glutamate concentration [42,43]. Among these, EAAT-1 and -2 are primarily essential for protection against excitotoxicity as was shown in EAAT gene knockout experiments in mice [44,45]. In addition to glutamate clearance, astrocytes also rapidly metabolize extracellular glutamate into glutamine with glutamine synthetase (GS) [46]. Glutamine can then be exported into the extracellular environment in close proximity to neuronal glutamine transporters. This cycle of glutamate import and glutamine export in astrocytes and neurons is collectively termed the glutamine/glutamate shuttle. A fine balance exists between the levels of glutamate released from the neurons, and glutamine, which is generated upon glutamate uptake by astrocytes and then transported to neurons. The glutamine/glutamate shuttle determines the resultant extracellular glutamate and an imbalance in this cycle leads to excess glutamate and excitotoxicity. In HIV-1 *gp120*-treated astrocytes, glutamate uptake was significantly reduced, and concurrently, a slow and consistent release of glutamate was observed [47].

During normal EAAT-2 functioning, glutamate and Na^+^ ions bind extracellularly and couple the transporter, inducing a conformational change, which releases glutamate into the cytosol. Intracellular K^+^ then binds inducing a second conformational change, which releases the K^+^ extracellularly and resets the transporter [38]. The Na^+^ and K^+^ electrochemical gradients function as the biochemical force powering import of extracellular glutamate against a several thousand-fold concentration gradient through EAAT-2 [48]. The functional capacity of EAAT-2 is sensitive to ATP levels, phosphorylation status, membrane translocation, and interactions with other ions [38]. Lowering ATP levels affects glutamate uptake, independent of EAAT-2 expression. Furthermore, reduced glutamate uptake during hyperthermia results from the depletion of ATP and reduced cotransport of Na^+^ and K^+^ ions across EAAT-2, thus diminishing its functional activity [6,49]. Thus, EAAT-2 activity is regulated at a functional level in response to the astrocytic energy state and microenvironment and contributes to the excitotoxic environment.

In addition to regulation of EAAT-2 activity, downregulation of EAAT-2 transcription also contributes to excitotoxicity. EAAT-2 transcription, studied by cloning of the EAAT-2 promoter, is potentially regulated by transcription factor binding elements, including NFAT [50]. Nuclear translocation of NFAT has been associated with forms of dementia [51]. NFAT recruitment inhibits EAAT-2 expression and subsequent extracellular glutamate accumulation activates astrocytic NFAT and inflammatory cytokine expression, suggesting an autoregulatory loop [51,52]. In addition to NFAT, other positive and negative regulators of EAAT-2 promoter activity include cyclic AMP (cAMP) and TNF-α, respectively [50]. Furthermore, both basal and enhanced EAAT-2 mRNA expression is regulated by a NF-κB-dependent mechanism [50]. In addition, EAAT-2 is highly regulated at the translational level due to a 5’ untranslated regions (UTR) larger then 300 nucleotides. Three EAAT-2 transcript variations have been identified, with 76, 310, and 1091 nucleotides, respectively, in their 5’ UTRs. The larger EAAT-2 transcripts require extracellular factor induction of transcription, and are also regulated by corticosterone, β-lactam antibiotics and retinol [53,54]. Therefore, astrocyte glutamate dysregulation may be dependent upon transcriptional and translational regulation of EAAT-2, in addition to transporter activity changes, when contributing to the excitotoxic microenvironment.

Astrocyte gap junctions also modulate glutamate homeostasis. Cx30/43 are major astrocytic gap junction proteins that form gap junctions responsible for buffering K^+^ levels and propagation of intracellular Ca^+2^ waves; which, in turn, regulate glutamate levels in the gap junction [55]. *In vivo* studies using conditional Cx43 knockouts, Cx30 complete or double knockouts, showed that increased EAAT-2 levels were observed when either Cx43 or Cx30 or both were knocked out. In contrast, EAAT-1 levels increased only in the double knock-out suggesting that astrocyte gap junctions regulated EAAT expression differentially [56]. In a rat model of microsphere embolism, dysregulation of basal glutamate concentrations was associated with downregulation of glutamate transporters [57]. Changes in basal glutamate levels have been shown to modulate mRNA and protein levels, and the kinetic properties of glutamate transporters [57]. The complexities of EAAT-2 regulation thus facilitate the fine balance between glutamate levels and synaptic homeostasis.

#### 3.1.2 Vesicular Glutamate Transporter-1

Recent investigations into gliotransmission, or astrocytic involvement in neurotransmission, have shown that astrocytes can exocytose neurotransmitters [41,58]. Intracellular Ca^+2^ oscillations observed in astrocytes induce glutamate release through VGLUT-1 localized on astrocyte processes in close proximity to the α-amino-3-hydroxy-5-methyl-4-isoxazolepropionate (AMPA) and *N*-methyl-D-aspartate (NMDA) receptors (NMDAR) in neurons, thus promoting communication with neurons and other CNS cells [59-61]. The mechanisms of glutamate release from astrocytes remains partially undefined, but inhibiting astrocyte exocytotic related proteins such as synaptobrevin-2, with clostridial toxin, prevents Ca^2+^-dependent release of glutamate [60]. In addition, Bezzi *et al. *show VGLUT-1 and -2, the vesicular soluble N-ethylmaleimide-sensitive factor attachment protein receptor (SNARE) protein, and cellubrevin are colocalized to vesicular organelles adjacent to neuronal glutamate receptors [62]. Upon activation of mGluRs, vesicles undergo Ca^2+^ and SNARE exocytic fusion accompanied by glutamate release [62]. Further studies, using VGLUT-1 overexpression constructs in astrocytes, indicate astrocytes require both cytosolic glutamate and VGLUT-1 to release glutamate by intracellular Ca^+2^ induction [63]. While astrocyte vesicular release of glutamate aids in neurotransmission and synaptic plasticity [59], increased expression of VGLUT-1 has been shown to cause excitotoxic neurodegeneration. The spontaneous vesicular release of glutamate *via *VGLUT-1 transports glutamate from intracellular to extrasynaptic space. Thus, despite the fact that VGLUT-1 is not primarily responsible for extracellular glutamate clearance or metabolism, it is important in glutamate release as a gliotransmitter [59].

#### 3.1.3 Metabotropic Glutamate Receptors

mGluRs are members of the group C family of inhibitory GPCRs activating [Ca^+2^]_i_ regulating protein expression and activation of an IP_3_ pathway, resulting in an intracellular release of Ca^+2^ and ATP [8,64] Ca^2+^ is an important secondary messenger that modulates cell proliferation, glutamate uptake and inflammatory mediator release. In astrocytes, glutamate-induced, increased [Ca^+2^]_i_ regulate astrocyte gliotransmitter release and thus modulate neuronal activity [65]. Activation of astrocyte mGluRs initiates the release of nerve growth factor and S-100β, which promote neuronal differentiation and survival. Additionally, astrocyte mGluRs are regulated by neuronal activity, further implicating the role of mGluRs during neuroprotection [33,66,67].

#### 3.1.4 Ionotropic Glutamate Receptors

Most fast excitatory synaptic transmission is mediated through glutamate acting on iGluRs. Two types have been reported, AMPA/kainate (non-NMDA) and the NMDAR, both directly gate ion channels. Recently, NMDAR mRNA and protein expression was demonstrated in primary human astrocytes. Immunocytochemistry confirmed NMDAR localization on GFAP-positive cells suggesting the likelihood of functional receptors. Indeed, NMDAR activation *via *glutamate opens cation channels ubiquitously. In astrocytes, glutamate activated the NMDAR, increasing [Ca^+2^]_i_ that was blocked by NMDAR-specific antagonists [68].

### 3.2 Synthesis and Metabolism of Glutamate

Two enzymes regulate glutamate metabolism and synthesis: glutaminase (GLS) and glutamine synthetase (GS), respectively. While GLS expression is low in astrocytes, GS is predominantly expressed in astrocytes and functional homeostasis of these enzymes is critical for neurotransmission and prevention of excitotoxicity.

#### 3.2.1 Glutaminase

The ability to exocytose glutamate is dependent upon the expression of phosphate-activated GLS, the mechanism of glutamate synthesis. GLS catalyzes the conversion of glutamine to glutamate and while it is not prominently localized to astrocytes, it is highly expressed in neurons of the CNS. GLS is regulated by ADP levels and is thus dependent on mitochondrial integrity. Elevated metabolism, decreased intracellular ROS and overall DNA oxidation are linked to GLS activity in both normal and stressed cells. Through GLS, modulation of ROS levels, p53 expression is also regulated, thus inhibiting genomic oxidative damage. Although scarce in astrocytes, GLS localizes predominantly between the mitochondrial membranes and active, phosphorylated GLS localizes specifically to the outer face of the inner mitochondrial membrane in astrocytes [9,10,69].

#### 3.2.2 Glutamine Synthetase

GS metabolizes nitrogen in an ATP-dependent reaction catalyzing the condensation of glutamate and ammonia to form glutamine and is predominately expressed in both microglia and astroglia. The glutamate amide group serves as a nitrogen source for the synthesis of glutamine pathway metabolites. Highly expressed in astrocytes during CNS stress, GS colocalizes with GFAP [70]. Recently Mongin *et al. *proposed a simplified, quantitative protocol to determine GS and GLS activity in astrocytes by measuring the mitochondrial conversion of glutamate to the tricarboxylic cycle intermediate α-ketoglutarate in oxidative deamination and transamination reactions. Using conditions optimized for astrocytes and appropriate pharmacological controls Mongin *et al*. conclude astrocytes express measureable amounts of GS and GLS indicating their contributions to synaptic plasticity and neurotransmission [10].

## 4 HIV-1 AND NEUROTOXICITY

HIV-1 infects the cells of the immune system, primarily CD4^+^ T cells and cells of the monocyte-macrophage lineage, and progressive immunodeficiency marks disease progression [71]. During acute HIV-1 infection virus replicates rapidly, leading to an abundance of virus in the peripheral blood. Despite the immune privileged status of the brain, HIV-1 has been linked to a number of neurological symptoms, even in the absence of confounding disease. HIV-1 penetrates the BBB, enters the CNS and causes varying degrees of neurocognitive impairment or HAND, characterized by cognitive, motor and behavioral disorders [71]. In parallel, as few common antiretroviral drugs adequately penetrate the CNS, creating a reservoir for virus until replication is reactivated within the CNS, in such, antiretroviral therapy (ART) has lessened the incidence, but not the prevalence of HAD [72]. Furthermore, information regarding extended, low level HIV-1 infection in the CNS is limited and the effects of ART on non-infected neural cells such as astrocytes are poorly studied [73]. One recent study reports that certain antiretroviral medications increase β-amyloid (Aβ) expression by neurons; Aβ plaque accumulation is a common feature of HIV-1 CNS infection [74]. Understanding the molecular and cellular mechanisms involved in response to low level, chronic viral expression in the brain along with the effects of ART on neural cells may further our understanding of HIV-1-associated neurodegeneration.

HAD, a clinical correlate of HIV-associated encephalitis (HIVE), often occurs during active, chronic HIV-1 infection of the brain. Approximately 15% of individuals infected with HIV-1 develop HAD, and approximately 30% to 60% of individuals develop a less severe form of minor cognitive motor disorders. During the pre-ART era, up to 25% of HIV-1-infected patients developed HAD as the first acquired immune deficiency syndrome (AIDS)-defining illness and early evidence of HIV-1 neuropathogenicity included HIV-1 in cerebrospinal fluid (CSF), abnormal neuroimaging, and the presence of HIV-1 in tissue obtained by brain biopsy [75]. CNS manifestations observed during HIV-1 viremia associated with seroconversion include meningitis, encephalitis, facial palsy, and peripheral nerve disorders; proposing HIV-1 neuroinvasion occurs early during infection.

Following HIV-1 penetration through the BBB, the virus productively infects and resides primarily in microglia and macrophages, playing a central role in neuroinflammatory disorders (reviewed by [76] and [77]). HIV-infected or immune-stimulated macrophages/microglia produce neurotoxins, and inflammatory mediators [78-80]. Macrophage- and microglia-derived factors including but not limited to: inflammatory chemokines and cytokines (such as, IL-1β and TNF-α), arachidonate and its metabolites (such as platelet-activating factor), free radicals, and viral proteins have been investigated in HIV-1-associated neurodegeneration (reviewed by [77]). While TNF-α, IL-1β, and IL-6 regulatory mechanisms suppress acute HIV-1 infection in microglia, the observations seen in chronically infected monocytic cell lines exacerbate disease [81].

While the BBB controls infiltration of substances/cells from the periphery, the HIV-1 disease process impairs the BBB, facilitating entry of HIV-1-infected monocytes as described by the classical Trojan horse hypothesis. HIV-1 proteins, *trans*-acting protein (*Tat*) and envelope glycoprotein (*gp)120*, and host proinflammatory cytokines and chemokines compromise the BBB, allowing free HIV-1 and transmigration of HIV-1-infected cells into the CNS [71]. Astrocytes interact with endothelial cells, as part of the gliovascular unit, and regulate the BBB physiology in attempt to protect its integrity [20]. Recently, Eugenin *et al.* describe a novel mechanism for bystander BBB toxicity, which is mediated by low numbers of HIV-1-infected astrocytes, amplified by gap junctions, and induces apoptosis of non-infected BBB cells [20]. Furthermore, Ju *et al*. demonstrate upregulation of both TNF-α and MMP-9 in response to HIV-1 *Tat* [82], a protein expressed by infected astrocytes, which can in turn affect other astrocytes.

Although acute HIV-1 replication is attenuated in the brain, HIV-1 mRNA expression in the CSF has been observed within eight days post-HIV-1 transmission [83]. The mechanisms underlying neurological and behavioral characteristics present in the first months following HIV-1 neuroinvasion are associated with HIV-1 *Tat* [84]. HIV-1 *Tat* is a key mediator of neurotoxicity promoting potentiation of glutamate and NMDA-triggered Ca^2+^, excitotoxic neuron apoptosis [85] and activation of GFAP expression in astrocytes [86]. Thus, reactive astrogliosis and glutamate excitotoxicity are both implicated in HIV-1 CNS neurodegeneration.

The abundance of astrocytes in the brain would make them an ideal target for HIV-1 infection and Hao *et al.* reported HIV-1 enters astrocytes through a receptor mediated endocytic pathway involving HIV-1 *gp120* in a concentration dependent manner [87]. Electron microscopy revealed HIV-1 virions in clathrin-coated pits of cytoplasmic vacuoles of astrocytes. While non-productive or latent infections of astrocytes were reported in the early decades of HIV/AIDS [88], many now believe that only ~2% of the brain astrocytes are likely infected by HIV-1 since astrocytes lack the ability to actively replicate HIV-1 and express viral regulatory genes [89].

In an artificial system, the WNT/β-catenin/transcription factor (TCF)-4 signaling pathway significantly inhibits HIV-1 replication in astrocytes; however, little is known about the direct mechanism. Knockdown of β-catenin and TCF-4 resulted in HIV-1 transcription in transiently and stably transfected cell lines with an integrated HIV long terminal repeat (LTR)-luciferase reporter construct. IFN-γ induced transcription of an antagonist of the β-catenin pathways, DKK1, in a signal transducers and activators of transcription (STAT)-3-dependent manner, which resulted in increased HIV-1 LTR activity through attenuated β-catenin signaling [90]. Costimulation of primary human astrocytes with METH and HIV-1* Tat* exacerbated β-catenin downregulation by greater than 50%. While METH alone had no effect, HIV-1 induced TCF-4 downregulation in astrocytes [91].

Despite limited infection of astrocytes by HIV-1, numerous studies, including ours demonstrate that HIV-1 virions do affect astrocyte inflammatory responses [92-94]. HIV-1 proteins such as HIV-1 *gp120* and HIV-1 *Tat* have also been shown to affect astrocyte function in multiple ways [95-98]. Furthermore, astrocytes are exquisitely sensitive to the proinflammatory cytokines present in the brain as a result of infection and many of the functional changes are amplified *via *autocrine and paracrine loops [18,93,99-106].

## 5 METH AND NEUROTOXICITY

METH addiction is a growing public health concern. METH users exhibit physical and psychological deficits, differing with acute versus chronic use. Acute METH administration depresses appetite, increases wakefulness and alertness resulting in more physical activity. Rapid, irregular heartbeat increases blood pressure and hyperthermia during acute METH exposure. Acute psychological effects include euphoria, increased energy levels, heightened libido and feelings of self-esteem and -confidence. Chronic use of METH contributes to anxiety, depression, aggressiveness, social isolation, psychosis, mood disturbances, and psychomotor dysfunction. Neuropsychological studies of chronic METH users detected deficits in attention span, working memory, and decision-making. These neuropsychiatric complications are related to drug-induced neurotoxic effects including damage to dopaminergic and serotonergic terminals (reviewed by [1]).

The high lipophilic nature of METH, attributed to the attached methyl group, allows it to cross the BBB and cellular membranes increasing the potentiation of neuropsychological effects. Furthermore, BBB compromise has been shown in the hippocampus, 24 hours post-METH injection, as measured by decreased TJ proteins, zonula occludens (ZO)-1, claudin-5 and occludin, and increased activity and immunoreactivity of MMP-9 [107]. Increased BBB permeability potentiates METH distribution to the brain and dysregulates dopamine (DA) concentrations. DA, a catecholamine, activates the psychological reward system, preventing normal functioning of the DA transporter (DAT). DAT dysregulation increases synaptic DA triggering the reward response. Psychological effects do not last long, as tolerance increases the euphoric feelings decrease. Chronic METH abuse eventually depletes the DA storage, attenuating the users euphoria.

Withdrawal symptoms occur with the abrupt disruption of METH use in approximately 90% of cases. Withdrawal enhances irritability, fatigue and intense craving for the drug linked to the recurrence of relapse. Zorick *et al. *investigated symptoms that appear during the first several weeks of METH abstinence, and how cravings for METH evolve into psychiatric symptoms persisting beyond a month of abstinence [108]. They found that while depressive and psychotic symptoms largely resolved within a week of abstinence, cravings did not decrease until the second week, and then continued at a reduced level to the fifth week. Low striatal DA functioning is associated with relapse in METH abstinent individuals and is the largest contributing factor in relapse [108]. Importantly, protracted abstinence leads to a reversal of DAT dysregulation in METH abusers and a clearer understanding of these mechanisms may lead to therapeutic prevention of relapse [108]. The direct mechanism of METH-induced DAT regulation is vague. Trace amine associated receptor (TAAR)1 is a stimulatory GPCR localized on membranes of dopaminergic neurons [109]. METH-induced activation of TAAR1 results in DAT internalization, reversal in the membrane and efflux of DA into the synapse. TAAR1 is a potential therapeutic target to effectively treating METH addiction.

Astrocytes have receptors located on their cell membranes for neurotransmitters and peptides, such as glutamate and DA, in addition to metabolizing enzymes making them susceptible to drug toxicity [110]. DA is metabolized by monoamine oxidase (MAO) resulting in the production of aldehydes and hydrogen peroxide, and astrocytes express both MAO-A and MAO-B, implicating their sensitivity to METH-induced DA fluctuations in the synapse [39]. Exogenous application of DA to astrocytes produces a dose-dependent increase in [Ca^+2^]_i_ stimulating transient Ca^+2^ uptake in mitochondria. DA metabolism produces hydrogen peroxide and activates lipid peroxidation, therefore activating phospholipase C, inducing IP_3_-dependent Ca^+2^ release from ER and increases in [Ca^+2^]_i_ by a receptor-independent mechanism. METH-induced DA in the synapse sensitizes astrocytes, making them more vulnerable to their environment inducing activation and increasing GFAP expression through mechanisms that still remain elusive [3]. METH-induced activation of astrocytes modulates glutamatergic transmission, and downregulate glutamate transporter and receptor expression and activity. It has been suggested that DA-induced oxidative stress, regulates the expression of EAAT-2 in astrocytes. ROS production interferes with the antioxidant enzyme equilibrium leading to oxidative stress-induced damage and excitotoxicity induced by EAAT-2 dysregulation. Brito *et al. *showed that while EAAT-2 mRNA and protein levels decreased in a dose-dependent manner to DA, glutamate uptake assays revealed a significant increase in astrocytic glutamate uptake capacity. Their rationale is that astrocytes release glutamate in a Ca^2+^-dependent manner, and as discussed, DA increases [Ca^+2^]_i_ in astrocytes leading to a phospholipase C, resulting in protein kinase (PK)C-dependent increase of glutamate synthesis and release further regulating extracellular glutamate concentrations [39]. Consistent with this observation, reduction in DA production attenuates METH toxic effects [111]. Nitric oxide (NO) insult in astrocytes affects EAAT-2 transcriptional regulation. NO insult causes dimerization of p65-c-rel, NF-κB subunits, and the reduced production of cAMP and the activation of the phosphatidylinositol 3 kinase (PI3K)/Akt pathway reduce the dimerization of NF-κB subunits, allowing for recuperation of NF-κB transcriptional activity responsible for regulation of EAAT-2 transcription [112].

Astrocytes exhibit a form of excitability and communication through changes in [Ca^+2^]_i_. The activation of glutamate receptors are coupled to various intracellular signaling cascades, including activation of PKC, Akt, cAMP/PKA/cAMP responsive element binding protein (CREB), and mitogen-activated protein kinase (MAPK) and Janus kinase (JAK) pathways [113]. Specifically activation of astrocyte mGluRs results in reduced intracellular cAMP activating Akt pathway and cAMP levels are mediating the activation of the PI3K/Akt pathway [112], suggesting an autoregulatory loop. The JAK/STAT pathway modulates astrogliosis and is exacerbated during METH exposure [113]. Herbert *et al. *sought out to identify pathways responsible for the glial response following injury. They found that GFAP upregulation occurred as early as six hours and reached a three-fold induction 48 hours following METH exposure [114]. METH downregulates extracellular regulated kinase and upregulates p38 MAPK. In the brain of HIV-infected METH users, there is induction of IFN-inducible genes, suggesting a dysregulation of the innate immune responses [115].

Extracellular stimuli, such as epidermal growth factor and other proinflammatory cytokines induce nuclear import of transcription factors and gene expression regulating the rewarding effects of METH [116]. METH decreases expression of CREB, modulating immediate early genes (IEG) expression *via *activation of the cAMP/PKA/CREB signal transduction pathway [117]. The METH-induced renormalization of the expression of several IEGs in rats chronically exposed to METH introduces a cellular mechanism that may be responsible for relapse in METH abstinent individuals.

## 6 COMBINED EFFECTS: METH, HIV-1 AND NEUROTOXICITY

Drugs of abuse exacerbate neurocognitive deficits associated with HIV-1, suppressing immunity *via *dysregulation of inflammatory mediator production, increasing viral replication in the CNS, in addition to increasing expression of the HIV-1 coreceptors CXCR4 and CCR5 in monocyte-derived dendritic cells [2]. HIV-infected brains show a METH-induced expression of IFN-inducible genes involved in the innate immune responses, secretion of pro- and anti-inflammatory cytokines and chemokines and ROS interfering with T-cell ability to control HIV-1 infection suggesting commonality of immune regulation [115].

Infected macrophages and microglia are responsible for productive replication of HIV-1 in the brain resulting in the release of viral proteins such as HIV-1 *gp120* and *Tat*. Although astrocytes do not actively contribute to the replication of HIV-1, studies show that cocaine results in astrocyte HIV-1 replication at the proviral DNA level measured by HIV-1 LTR-R/U5 amplification, and secretion of HIV-1 *p24* antigen [118,119]. These viral proteins bind to DAT and impair its function initiating a feedback loop of increasing synaptic DA concentrations further increasing HIV-1 replication and inflammatory mediators production [120]. In addition, METH increases DA concentration in the synapse, modulating HIV-1 neurotoxicity. A mutant strain of mice (VMAT2 LO) was utilized to show exacerbated loss of DAT and tyrosine hydroxylase along with enhanced astrogliosis following METH insult to the striatum [111]. The direct neurotoxic effects of METH aggravate HIV-1-associated neuronal injury, increasing the combined effects of proinflammatory mediators, ROS, and excitotoxicity.

A common characteristic present with METH abuse and HIV-1 infection in the brain is the generation of ROS. [Ca^+2^]_i_ levels in astrocytes are responsible for excitability and communication stimulated by neuronal synaptic activity. Astrocytes sensitivity to DA- and glutamate-induced intracellular Ca^+2^ flux is responsible for deficiency in cellular redox homeostasis [121]. Nuclear factor-erythroid 2-related factor 2 (Nrf2) is an important regulator of redox homeostasis and its binding activity to antioxidant response elements on genes modulates their transcription during ROS induction. Exposure of hydrogen peroxide to astrocytes strongly activates astrocytic Nrf2/antioxidant response element dependent gene expression [122]. Astrocyte upregulation of metallothionein, a family of neuroprotective proteins against DA quinone neurotoxicity, is induced by Nrf2 nuclear translocation, suppressed by DAT inhibitors. Results suggest excessive DA uptake *via *astrocytic DAT mediates metallothionein expression promoting neuroprotection [123]. METH-induced astrogliosis is initiated during Nrf2 deficiency, indicated by increased striatal expression of GFAP [124]. Nrf2 inhibits HIV-1 *Tat*-induced HIV-1 LTR transactivation in MAGI cells [125] and prevents increases of TNF-α mRNA and IL-15 protein expression in GFAP positive cells. HIV-1 *Tat*-enhanced cellular expression of Nrf2 at transcriptional and translational levels activating the expression of antioxidants, indicating that Nrf2 has a key role in primary neuroprotection.

### 6.1 Blood Brain Barrier

The BBB functions to separate circulating blood from brain extracellular fluid, preventing materials present in the blood from entering the brain. As discussed earlier, HIV-1 neuroinvasion occurs as a result of HIV-1-infected monocytes crossing the BBB and infecting resident microglia, secreting proinflammatory mediators and generating ROS. METH elicits inflammation contributing to diminished integrity of the BBB leading to rapid HIV-1 neuroinvasion, therefore, increasing the risk of developing HAND.

TJ proteins are an integral part of this microenvironment. Rho kinase phosphorylates TJ proteins, claudin-5 and occludin in brain tissues of patients diagnosed with HIVE leading to diminished BBB integrity [126]. As discussed previously, HIV-1 *Tat* has been shown to disrupt TJs associated with astrocytes along the barrier, increasing the permeability of the BBB. Astrocytes treated with HIV-1 *Tat *showed increased MMP-9 mRNA and protein levels, as meditated by NF-κB and MAPK [82]. METH additionally decreases occuldin protein expression in a dose-dependent manner [127]. Furthermore, HIV-1 *gp120* in conjunction with METH alters BBB permeability by modulating TJ protein expression; as ZO-1, junctional adhesion molecule-2, occludin, claudin-3 and claudin-5 impairments were involved Rho-A activation [128]. Both METH and HIV-1 *gp120* independently and concurrently modulate TJ protein expression. Furthermore, synergistic effects of METH and HIV-1 increase the activity of MMPs, by increasing the release of MMP-1 and MMP activator from human neuron and human astrocyte cultures [129].

### 6.2 Astrogliosis

An important astrocyte characteristic is the vigorous response to an array of neurological insults, including METH and HIV-1. Various signaling pathways and IEG mediate the rapid reactive astroglial responses, shown by GFAP upregulation, proliferation, and morphology changes. GFAP is widely used as an astrocytic marker and is a robust indicator for reactive astrocytes, thus molecular mechanisms of increased GFAP expression are important for the study the indirect effects of METH/HIV-1-induce neurotoxicity *via *astrocytes [86].

While astrocytes have limited entry of HIV-1 and restricted HIV-1 viral gene expression, a common characteristic is changes in the GFAP expression patterns. Studies utilizing an adapted retrovirus-mediated gene delivery system and an established human glioblastoma U373.MG cell line derivative expressing HIV-1 *Tat* [130] show HIV-1 *Tat *produced successful induction of GFAP in a human glioblastoma U373.MG cell line. Zhou *et al. *showed HIV-1 *Tat* upregulates GFAP in astrocytes, which is induced at the transcriptional level through *cis*-acting elements of early gene response 1; and that GFAP regulates HIV-1 *Tat* neurotoxicity [131]. METH also causes upregulation of GFAP, shown by *in vivo* and *in vitro* studies; however, acute METH use has been shown to markedly increase activated astrocytes, resulting in PKC phosphorylation, thereby developing METH-induced desensitization [116]. In the striatum of chronic METH users who died of drug intoxication, both GFAP and S-100β were visualized by immunohistochemistry. The number of GFAP-positive astrocytes was not significantly higher as compared to S-100β-positive cell density, demonstrating the absence of reactive gliosis in the striatum of chronic METH users, *i.e.*, those who did not abstain for prolonged periods from METH use. One study addressed the percentage of activated astrocytes in brains of chronic METH users, who died as a result of METH intoxication, in comparison to non-users. In the study, investigators immunohistochemically studied microglial and astroglial activation and found that GFAP-positive cell density was not significantly different between cohorts; however, the number of GFAP-positive cells was elevated in chronic users as compared to non-users. Thus, most astrocytes from chronic METH abusers had normal morphology. GFAP-positive cells rarely exhibited hypertrophic cell bodies, shortening of cytoplasm processes or nuclear enlargement. The study suggests that astrocyte activation, observed during acute METH administration, *in vivo*, is attenuated during a chronic METH exposure [132]. However, a separate recent report suggests that few GFAP-positive astrocytes are ever observed in postmortem tissue measured by immunocytochemistry probing for GFAP-positive astrocytes [91]. Stadlin *et al. *observed an increase in reactive astrocytes 48 hours post-METH treatment *in vivo*, as measured by western blot analysis of GFAP and vimentin in brain lysates of mice, suggesting that the decrease in activated astrocytes may be attributed to the analysis of post-mortem tissue in comparison to living tissue [133]. Increased Myo-inositol, an *in vivo* marker used to study astrocyte activation measured by neuroimaging is indicative of astrocyte activation [134]. Data from HIV-1+/METH+ subjects describe an increase in the levels of myo-inositol compared to HIV-1+/METH- and HIV-1-/METH+ [134,135].

The GFAP promoter contains *cis*-acting elements that are responsive to several growth factors and cytokines, and contains binding sites for transcription factors such as, activator protein (AP)-1, specificity protein-1, and NF-1, and modulates expression of CCL2 [131]. As discussed, GFAP induction occurs at the transcriptional level through *cis*-acting elements of the early growth response (Egr)-1 within its promoter [86]. Several IEG members are associated with METH/HIV-1-induced astrogliosis. HIV-1 *Tat* exposure in human astrocytes results in elevated platelet derived growth factor expression levels, regulated by the downstream transcription factor Egr-1, leading to increased proliferation and release of CCL2 and IL-1β [136] and reported to upregulate GFAP expression. METH-induced DA fluxes lead to differential expression of IEGs. Acute METH injections caused delayed induction of the Egr family of transcription factors in a concentration-dependent fashion [137]. Chronic METH treatment reduces expression of AP-1, Erg1–3, and Nr4a1 transcription factors below control levels and acute injection of METH increased c-fos, fosB, fra2, junB and Egr1–3 mRNA levels in the striatum [117,138]. Egr-1 patterns during acute and chronic METH abuse suggest a correlation between astrocyte activation and Egr-1 expression.

### 6.3 Astrocyte-Induced Excitotoxicity

**Fig. (2):**
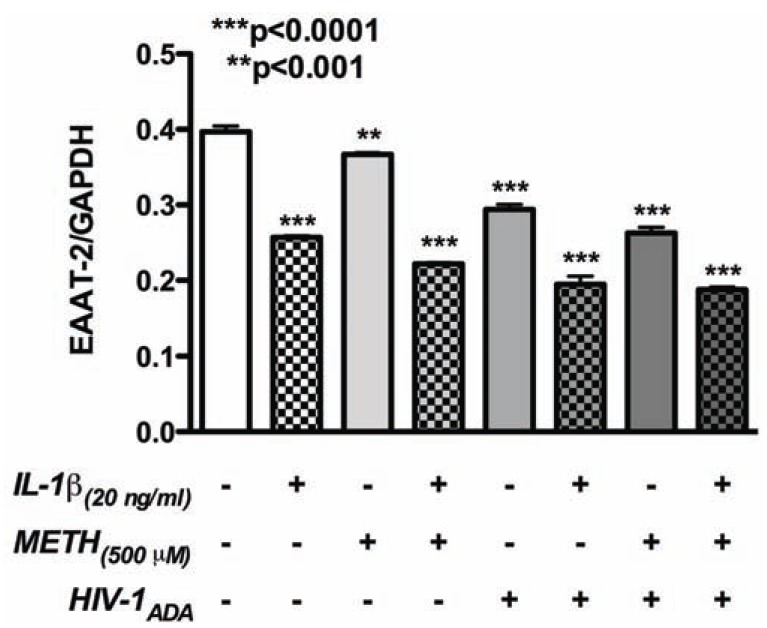
**METH, HIV-1, and inflammatory mediators alter EAAT-2 mRNA expression**. Primary human astrocytes were treated with METH (500 µM), HIV-1_ADA_, and IL-1β (20 ng/mL) for 24 hours. Real-time PCR was performed to determine the mRNA expression patterns of EAAT-2. METH, HIV-1_ADA_ and IL-1β independently led to significant decreases in EAAT-2 mRNA levels. A combination of stimuli further attenuated EAAT-2 mRNA levels indicating additive/synergistic effects. Astrocyte RNA was extracted with Trizol reagent (Life Technologies Corp., Carlsbad, CA) and reverse-transcribed into cDNA as per the manufacturer’s instructions (Life Technologies). TaqMan^®^ 5' nuclease gene expression assays for EAAT-2 (Life Technologies, C/N: hs00997364_m1) and glyceraldehyde phosphate dehydrogenase (GAPDH; C/N: 4310859) were performed using a StepOnePlus system (Life Technologies). The reactions were carried out at 48°C for 30 min, 95°C for 10 min, followed by 40 cycles of 95°C for 15 s and 60°C for 1 min. Gene expression was expressed as mean ± standard error of mean (SEM) of triplicates and are representative of 3 astrocyte donors tested as biological replicates. Multiple additional astrocyte donors were examined in smaller subsets of treatment conditions in several other experiments. In each experiment, individual conditions were tested with a minimum of triplicates. Gene expression was compared by one-way analysis of variance followed by Newman-Keuls multiple comparisons post-tests (Prism 5.0, GraphPad software, La Jolla, CA, USA).

Excitotoxicity is common morbidity associated with METH abuse and HIV-1 infection. Mechanisms contributing to excitotoxicity include downregulation and dysfunction of glutamate transporters, glutamate receptors, and glutamate synthesizing and metabolizing enzymes in astrocytes; however, the mechanisms that dictate these functions are unknown. Studies of astrocyte contributions to excitotoxicity have largely been concentrated on EAAT-2 regulation. EAAT-2 downregulation in primary human astrocytes in response to HIV-1 infection, METH abuse, and in the presence of inflammatory mediators is widely accepted; synergy in these pathways is unclear. Our results show EAAT-2 mRNA attenuation during short-term exposure to a combination of stimuli (Fig. **2**). METH, HIV-1 and IL-1β, a prototypical proinflammatory cytokine, led to significant decreases in EAAT-2 mRNA levels; demonstrating combined effects on glutamate regulation. NF-κB is an important transcriptional regulator of the EAAT-2 promoter and under specific conditions may act as a transcriptional repressor [139]. Astrocyte NF-κB transcriptional activity is initiated by activation of the PI3K/Akt pathway, mediated by cAMP levels [112], increasing EAAT-2 [140]. Additionally fragments of Ntab, partial sequences of non-coding RNA located in the 3’ UTR of EAAT-2 gene, act as enhancers for EAAT-2 transcription [141], but Ntab regulation during METH/HIV-1-induced neurotoxicity has not been investigated. Translational regulation of EAAT-2 is influenced by methylation, which has been shown to result in silencing of EAAT-2 in astrocytes [142]; but again, has not been investigated under METH/HIV-1 conditions.

Increased ROS/NOS is a common result of METH and HIV-1 exposure in the brain and is suggested to regulate EAAT-2, mGluRs, VGLUTs and glutamate metabolic enzymes. A previous study suggested that METH-induced chronic stress initiates the vesicular release of glutamate, thus directly contributing to the increased extracellular glutamate concentrations [143].

Enzymes responsible for glutamate metabolism are dysregulated during METH/HIV-1-induced neurotoxicity. GS was shown to be upregulated during an inflammatory CNS state [69] or when exposed to inflammatory cytokines [144]. Studies addressing the molecular mechanisms associated with astrogliosis during METH abuse [133] demonstrate rapid depletion of GS in METH-treated astrocytes. The regulation of this enzyme during METH abuse has been studied *in vitro*. Stadlin *et al. *concluded that the amount of GS was depleted more rapidly in response to METH in striatal astrocytes, by 50% at 8 hours, and in cortical astrocytes, by 48% at 48 hours. Therefore suggesting that astrocytes of the dopaminergic system are more sensitive to METH-induced GS expression and METH-induced oxidative stress led to the significant decrease in GS and increased levels of intracellular glutamate [133]. HIV-1 *gp120* reduced glutamine concentrations and GS expression with N-acetylcysteine effectively counteracting the effects of HIV-1 *gp120* [145].

Recent studies in human mononuclear phagocytes (microglia and macrophages) regarding GLS and HIV-1 infection report the significant increase in glutamate production following HIV-1 infection is dependent on GLS; suggesting increased extracellular glutamate in the synapse contributes to excitotoxicity. Utilizing siRNA to inhibit GLS synthesis significantly reduced extracellular glutamate levels [146]. GLS, a mitochondrial protein, is released into the cytosol and extracellular space following infection, rapidly catalyzing the reaction of extracellular glutamine to glutamate. Although not a direct mechanism observed in astrocytes, the process is taking place in the extracellular milieu, in close proximity to astrocytes, which are experiencing limited EAAT-2 expression to compensate for these events [147]. GLS dysregulation in microglia is proposed to enhance neurotoxicity, suggesting that HIV-1 in the CNS increases glutamate concentrations *via *dysregulation of enzymes, transporters and receptors [146].

## 7 CROSSTALK

Neuronal activity participates in dynamics of the astrocyte glutamate transporter [33]. Astrocyte EAAT-2 expression *in vitro* is enhanced when cocultured with neurons [33,148] *via *NF-κB subunits, p65 and p50, binding and enhancing the activity of the EAAT-2 promoter and dominant-negative inhibitors of NF-κB signaling completely blocked neuron-dependent activation of a NF-κB reporter construct [149]. Exogenous expression of the NF-κB subunits p65 and/or p50 induced EAAT-2 expression and EAAT-2-mediated transport activity [149]. Addition of neuron-conditioned media or separation of neurons from astrocytes with a semipermeable membrane has likewise demonstrated the importance of a coculture system [150]. Ghosh *et al.* further investigated the direct mechanism by which neurons induce EAAT-2 expression in astrocytes. Results from several of their experiments provide evidence that NF-κB signaling in astrocytes contributes to the neuron-induced activation of glial glutamate transporter (GLT)-1, *i.e*. EAAT-2 in mice, with NF-κB subunits interacting with specific sites in the GLT-1/EAAT-2 promoter. NF-κB signaling was studied utilizing transgenic mice that express astrocyte enhanced green fluorescent protein (eGFP) controlled by a bacterial artificial chromosome (BAC) with DNA surrounding the GLT-1/EAAT-2 gene, (BAC GLT-1 eGFP mice). Using NF-κB reporter constructs introduced into astrocytes, Ghose *et al*. determined that overlaying neurons increased reporter activity, and using inhibitors for NF-κB signaling (IκBα-SR and IκBα-ΔN) attenuated neuron-induced expression of eGFP. Exogenous expression of NF-κB subunits, p50 and p65, increased eGFP expression in astrocytes, and binding of these subunits was detected *in vivo* and *in vitro*. Mutating the NF-κB binding sites in the GLT-1 promoter region eliminated neuron-induced activation of the GLT-1 promoter. These conclusions suggest that neuronal activity also regulates astrocyte EAAT-2 [149].

Investigations addressing the physical contact of astrocytes and neurons in modulating astrocyte EAAT-2 expression show, that in microfluidic chambers, axons located on the presynaptic termini induce glutamate transporter expression due to astrocyte and neuronal membrane contact [151]. Astrocytes physically adhere to neurons *via *integrin receptors initiating activation of PKC [152]. Astrocytic contact induces excitatory synaptogenesis in the neuron upon METH exposure causing phosphorylation of PKC in both cell types and astrocyte activation [152]. Data suggest METH-induced, PKC-dependent activation of glutamatergic transmission may contribute to psychological dependence [153].

HIV-1 and METH indirectly affect glutamate receptor functioning in neurons, typically through direct effects in astrocytes. Crosstalk between astrocytes and neurons is critical in regulating glutamate receptors and transporters in both cell types. One recent report shows that IL-1β-activated astrocytes release soluble factors; such as, CXCL8, CCL2, MMP-1 and MMP-7, in addition to NMDAR-like agonists. Conditioned media from human astrocytes, with and without IL-1β stimulation, was added to oocytes that were injected with NR1a/NR2B, subunits of the NMDAR. Results indicate that the release of soluble factors that are similar to NMDAR agonists from IL-1β-treated astrocytes, leads to activation of the NMDA subunits, suggesting a mechanism of crosstalk associated with IL-1β/virus-treated astrocytes and NMDA activation in neighboring neurons. Previous studies show that NMDA antagonists attenuate the neurotoxic effects associated with HIV-1. IL-1β-stimulated/or virus-infected astrocytes have been shown to release quinolic acid, a byproduct of DA metabolism, and also produced ROS, which may serve as another potential the link between the comorbidity of METH and HIV-1 in astrocytes [154].

## CONCLUDING REMARKS

In the post-ART HIV-1 era, multiple cofactors have emerged, contributing to the long-term implications of ART and the prolonged lifespan of infected patients. While drug abuse has always been a consideration in influencing multiple aspects of HIV-1 disease progression, now even more so than before, it is emerging as a significant threat with the ever-increasing prevalence of drug abuse by patients. The focus of this review is METH and its specific contributions on the neurological implications in HIV-1-infected patients. Specifically, the culprit of ‘excitotoxicity’; a result of imbalance in a critical neurotransmitter, glutamate, is examined in the context of METH and HIV-1 combined injury. The dysregulation of glutamate is implicated in multiple neurodegenerative disorders and is neither specific to HIV-1 nor METH abuse; however, the combined neurotoxicity of both on the CNS and their mechanistically complimentary and/or synergistic effects influence the outcome of both ailments. Interestingly, excitotoxicity in the brain is a resultant of dysregulated mechanisms in astrocytes affecting neurons, when neither the astrocytes nor the neurons are direct targets of viral infection. Typically, glutamate imbalance is an outcome of dysregulation of astrocytic glutamate transporters and other complimentary pathways in both astrocytes and neurons. Astrocytes regulate extracellular glutamate concentrations through EAAT-2 activity and, although there is no direct infection and replication of HIV-1 in astrocytes, nor a clear understanding of the mechanism of METH action in astrocytes, METH and HIV-1 independently downregulate EAAT-2 expression. METH/HIV-1-induced BBB impairment and astrogliosis are also evidence of METH/HIV-1-induced effects in astrocytes. Thus, several lines of evidence demonstrate common outcomes in METH and HIV-1 in the CNS; yet, the combined mechanisms of injury and mechanistic synergy remain to be elucidated. Clearly, further investigation into the comorbidity pathways of METH and HIV-1 in the CNS are necessary. We propose that common mechanisms culminating upon glutamate imbalance through regulation of astrocyte glutamate transporters, is an area that warrants further insight. Selective drugs that target glutamate transporters and enhance their expression may serve as future therapeutic targets not only in the setting of METH/HIV-1, but will prove vital for multiple neuroinflammatory/neurodegenerative disorders which involve excitotoxicity as the mechanism leading to neurological malfunction.

## Figures and Tables

**Fig. (1):**
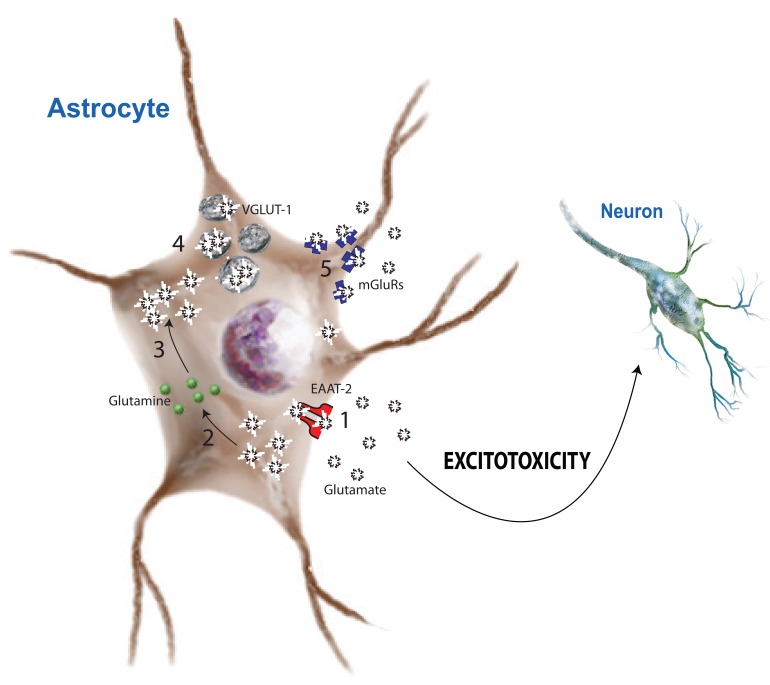
**Mechanisms of astrocyte-mediated glutamate regualtion**. Astrocytes are intricately involved in glutamate regulation through several mechanisms. Excitatory amino acid transporter-2 (1, EAAT-2) is responsible for uptake of extracellular glutamate into the cell, where intracellular glutamine synthetase (2, GS) catalyzes the reaction forming glutamine from glutamate. Glutaminase (3, GLS) catalyzes the reaction to convert glutamine to glutamate where astrocytes can package it into vesicular glutamate transporters (4, VGLUT-1). The expression of functional metabotropic glutmate receptors (5, mGluRs) allows glutamate signaling leading to increased intracellular calcium levels.

## ABBREVIATIONS

AMPA: = α-amino-3-hydroxy-5-methyl-4-isoxazolepropionate
AIDS: = Acquired immune deficiency syndrome
AP: = Activator protein
ARE: = Antioxidant response element
ART: = Antriretroviral therapy
Aβ: = β-amyloid
BAC: = Bacterial artificial chromosome
BBB: = Blood brain barrier
Ca^+2^: = Calcium
CREB: = cAMP responsive element binding protein
CNS: = Central nervous system
CSF: = Cerebrospinal fluid
Cx: = Connexin
cAMP: = Cyclic AMP
COX: = Cyclooxygenase
DAT: = Dopamin transporter
DA: = Dopamine
Egr: = Early growth response
eGFP: = Enhanced green fluorescent protein
EAAT: = Excitatory amino acid transporters
ECM: = Extracellular matrix proteins
GPCR: = G-protein coupled receptors
GFAP: = Glial fibrillary acidic protein
GLT: = Glial glutamate transporter
GLS: = Glutaminase
GS: = Glutamine synthetase
HIV-1 *gp120*: = HIV-1 envelope glycoprotein
HIV-1 *Tat*: = HIV-1 *trans*-acting protein
HAD: = HIV-associated dementia
HIVE: = HIV-associated encephalitis
HAND: = HIV-associated neurocognitive disorders
HIV-1: = Human immunodeficiency virus
IEG: = Immediate early genes
IP_3_: = Inositol phosphate
IFN: = Interferon
IL: = Interleukin
[Ca^+2^]_i_: = Intracellular calcium concentration
iGluR: = Ionotropic glutamate receptor
JAK: = Janus kinase
LTR: = Long terminal repeat
LTP: = Long-term potentiation
MMP: = Matrix metalloproteinases
mGluRs: = Metabotropic glutamate receptors
METH: = Methamphetamine
MAPK: = Mitogen-activated protein kinase
MAO: = Monoamine oxidase
gfa2: = Mouse GFAP genes
NMDA: = *N*-methyl-D-aspartate
NMDAR: = *N*-methyl-D-aspartate receptors
NO: = Nitric oxide
NF: = Nuclear factor
NFAT: = Nuclear factor of activated T-cells
Nrf2: = Nuclear factor-erythroid 2-related factor 2
PI3K: = Phosphatidylinositol 3 kinase
K^+^: = Potassium
PK: = Protein kinase
ROS/NOS: = Reactive oxygen/nitrogen species
STAT: = Signal transducers and activators of transcription
siRNA: = Small interfering RNA
Na^+^: = Sodium
SNARE: = Soluble N-ethylmaleimide-sensitive factor attachment protein receptor
TJ: = Tight junction
TIMPs: = Tissue inhibitors of MMPs
TAAR: = Trace amine associated receptor
TCF: = Transcription factor
TNF: = Tumor necrosis factor
UTR: = Untranslated regions
VGLUT: = Vesicular glutamate transporters
ZO: = Zonula occludens
